# Prognostic factors for venous thrombosis in patients with peripherally inserted central catheters: Protocol for a systematic review and meta-analysis

**DOI:** 10.1097/MD.0000000000021037

**Published:** 2020-07-10

**Authors:** Yanling Gao, Xiaoyi Fan, Jie Han

**Affiliations:** Department of Integrated Chinese and Western Medicine, Henan Cancer Hospital & Zhengzhou University Cancer Hospital, Zhengzhou, China.

**Keywords:** meta-analysis, peripherally inserted central venous catheters, prognostic factor, protocol, venous thrombosis

## Abstract

**Background:**

Peripherally inserted central catheters (PICCs) has become increasingly popular in clinical practice because of the ease and safety of insertion and lower cost-effectiveness. The precise incidence and risk of PICC-related venous thrombosis is important to be verified in the context of growing PICC use and an understanding of the risk of venous thrombosis is an important cost and patient safety question.

**Method:**

We will search seven electronic databases including the Cochrane Library, MEDLINE, EMBASE, Chinese BioMedical Database, China National Knowledge Infrastructure, Chinese VIP and Wangfang Database regardless of publication date or language. All studies with prognostic factor analysis will be included if they recruited participants with PICC. Primary outcomes will include venous thrombosis. The risk of bias will be assessed by 2 authors using quality in prognostic studies tool. If possible, a meta-analysis in fixed or random effects model will be conducted by R-3.5.1 software, otherwise a narrative synthesis will ensue focusing on prognostic factors. The confidence in cumulative evidence will be assessed by Based on the Grading of Recommendations Assessment, Development and Evaluation.

**Results:**

The aim of this study is to retrieve, appraise and summarize the clinical evidence of risk assessment for PICC-related venous thrombosis.

**Conclusions:**

This study will assess the precise incidence and risk of venous thrombosis in patients with PICC and provide references for establishing relevant assessment tools.

**Ethics and dissemination:**

This study is a protocol for systematic review and meta-analysis of prognostic factors for venous thrombosis in PICC patients. This review will be published in a journal and disseminated in print by peer-review.

## 1 Introduction

Peripherally inserted central catheters (PICCs) has become increasingly popular in clinical practice because of the ease and safety of insertion and lower cost-effectiveness compared with other central venous catheters.^[1,2]^ The use of peripherally inserted central catheters is an alternative for medium and long-term vascular access in more than 2.5 million people worldwide annually.^[3]^ Furthermore, the proliferation of experienced nurse-led PICC teams has made PICC use convenient and accessible in medical practice,^[1,4–6]^ for example, intravenous nutrition, and radio-chemotherapy for patient with cancer. However, PICCs are associated with important complications despite its various of benefits.

PICC-related complications are common and important problem in clinical application. Venous thrombosis is 1 of the most common complications in PICC patients with the incidence of 5% to 20%.^[7,8]^ PICC-related venous thromboembolisms not only interrupt treatment, but also in-crease cost, morbidity, and mortality. Existing studies reported that PICC-related venous thrombosis is associated with the risk factors including age, coagulation, disease history and PICC instruments.^[9–13]^ However, it is unfortunate that the risk of PICC-related venous thromboembolism is uncertain.

In summary, the precise incidence and risk of PICC-related venous thrombosis is important to be verified in the context of growing PICC use and an understanding of the risk of VT is an important cost and patient safety question. Hence, we will retrieve, appraise and summarize the clinical evidence of risk assessment for PICC-related venous thrombosis and provide references for establishing relevant assessment tools.

## 2 Methods

### 2.1 Objectives and registration

This review will be to assess and summarize the available evidence of prognostic factors for venous thrombosis in patients with peripherally inserted central catheters. This review protocol is adhere to the Preferred Reporting Items for Systematic Reviews and Meta-Analyses Statement(PRISMA-P)^[14]^ and registered in the OSF platform (https://osf.io/registries) with a registration number 10.17605/OSF.IO/ATJHB.

### 2.2 Inclusion criteria

#### 2.2.1 Types of studies

All studies with prognostic factor analysis will be included in this systematic review regardless of type of study, publication status and language.

#### 2.2.2 Types of participants

Participants with peripherally inserted central catheters, who were 18 years or older, will be included regardless of their age, sex, or race.

#### 2.2.3 Types of exposure

The exposure of interest will be peripherally inserted central catheters.

#### 2.2.4 Types of outcome measures

Primary outcomes will be venous thrombosis.

### 2.3 Search methods for the identification of studies

We will search 7 electronic databases including the Cochrane Library, MEDLINE, EMBASE, Chinese BioMedical Database, China National Knowledge Infrastructure, Chinese VIP Information and Wangfang Database regardless of publication date or language. We will conduct different strategies for 7 electronic databases based on symptom terms (venous thrombosis, deep vein thrombosis, pulmonary embolism, and venous thromboembolism), exposure terms (peripherally inserted central catheters and PICC).

### 2.4 Data collection

#### 2.4.1 Selection of studies

**Figure 1:**
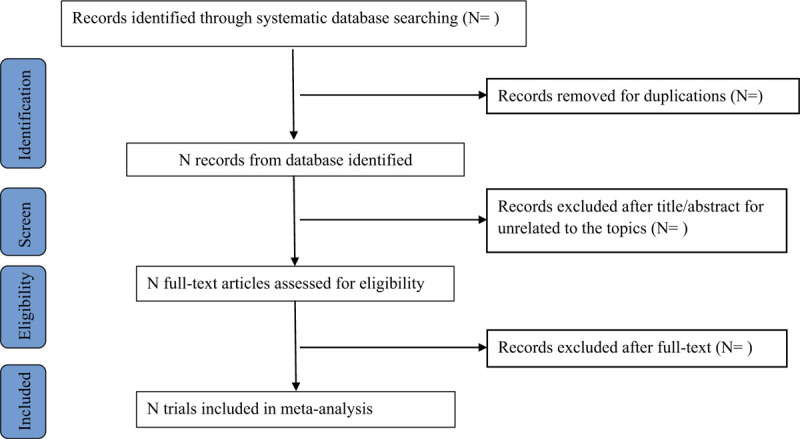
Flow chart of study selection.

All potentially eligible articles will be retrieved and organized in the Endnote X9 reference manager software and duplicate publications will be deleted. Two review authors (YLC and JH) will independently scan the titles and abstracts of all potentially eligible articles identified from electronic databases. Full-text articles will be scanned for all potentially relevant articles. If there is any disagreement on the selection of articles, they will be discussed with the third author (XYF). The PRISMA flow chart is displayed in Figure 1.

#### 2.4.2 Data extraction and management

Two review authors (YLC and JH) will independently extract the relevant information using a standard data extraction table. Information will include publication of year, author, participants, intervention, control, duration of intervention, outcomes and methodological characteristics. If disagreement is reached, the opinion will be resolved by discussion by arbiter (XYF).

### 2.5 Assessment of the risk of bias

Two authors (YLC and JH) will independently assess the risk of bias using the Quality in Prognostic Studies (QUIPS) tool.^[15]^ Six potential items will be assessed: study participation, study attrition, prognostic factor measurement, outcome measurement, study confounding and statistical analysis and reporting. The judgments of evaluated domains will include yes, partly, no and unsure. If disagreement is reached, the opinion will be resolved by discussion by arbiter (XYF).

### 2.6 Assessment of reporting biases

Funnel plots will be used to assess the potential for small study bias if there are sufficient studies. Asymmetry of funnel plots will suggest possible small study effects and the results will be explained cautiously.^[16,17]^

### 2.7 Data synthesis and statistical analysis

Effect sizes will be summarized as risk ratios or odds ratio with their 95% confidence intervals by R-3.5.1 software. Subject to homogeneity of prognostic factors, outcomes and timing of outcome, a meta-analysis will be performed. The Higgins *I*^2^ statistic will be used to examine heterogeneity for quantifying inconsistency in the included studies. Standard meta-analysis in random effects model will be conducted if *I*^2^ >0.5. For insufficient or missing data, we will contact the authors by e-mail or phone as much as possible. All analysis will be performed based on intent-to-treat principle.

#### 2.7.1 Subgroup and sensitivity analysis

Considered of possible significant heterogeneity, subgroup analysis will be performed in order to explore the differences in the age, sex, methodological quality, type of studies and race/ethnicity. To assess the robustness of the data synthesis, sensitivity analysis will be carried out whenever possible.

#### 2.7.2 Confidence in cumulative evidence

We will assess the level of evidence on venous thrombosis by Grading of Recommendations Assessment, Development and Evaluation.^[18]^ The quality of the body of evidence will be assessed based on 5 factors, including study limitations, effect consistency, imprecision, indirectness, and publication bias. The assessments will be categorized as high, moderate, low, and very low quality.

## Author contributions

FXY and GYL developed the study protocol. GYL and HJ developed the search strategy with supervision of FXY. GYL and HJ will scan the included studies, extract the data and assess the risk of bias. GYL and HJ will perform data analysis with supervision of FXY. All authors (GYL, FXY and HJ) will contribute to data interpretation. GYL, FXY and HJ drafted and revised the manuscript. All authors have read and approved the final version of the manuscript.

**Conceptualization:** Yanling Gao, Xiaoyi Fan.

**Data curation:** Yanling Gao, Xiaoyi Fan, Jie Han.

**Formal analysis:** Yanling Gao, Xiaoyi Fan, Jie Han.

**Funding acquisition:** Yanling Gao.

**Methodology:** Yanling Gao, Xiaoyi Fan, Jie Han.

**Software:** Yanling Gao, Jie Han.

**Supervision:** Xiaoyi Fan.

**Writing – original draft:** Yanling Gao, Xiaoyi Fan, Jie Han.

**Writing – review & editing:** Yanling Gao, Xiaoyi Fan, Jie Han.
